# Variation in Use of Lung Cancer Targeted Therapies Across State Medicaid Programs, 2020-2021

**DOI:** 10.1001/jamanetworkopen.2022.52562

**Published:** 2023-01-25

**Authors:** Thomas J. Roberts, Aaron S. Kesselheim, Jerry Avorn

**Affiliations:** 1 Program On Regulation, Therapeutics, And Law (PORTAL), Division of Pharmacoepidemiology and Pharmacoeconomics, Department of Medicine, Brigham & Women’s Hospital, Boston, Massachusetts; 2 Department of Medicine, Massachusetts General Hospital, Boston; 3 Department of Medical Oncology, Dana-Farber Cancer Institute, Boston, Massachusetts; 4 Harvard Medical School, Boston, Massachusetts

## Abstract

**Importance:**

Targeted therapies for *EGFR* (OMIM 131550)– and *ALK* (OMIM 105590)–altered metastatic non–small cell lung cancer (NSCLC) substantially improve outcomes for some patients. However, use of these therapies is lower among Medicaid patients, and access to oncology care varies across state Medicaid programs. Evidence is lacking on how use of targeted therapies for metastatic NSCLC varies across state Medicaid programs.

**Objectives:**

To characterize state-level variation in the use of targeted therapies among Medicaid patients with metastatic NSCLC and to describe factors associated with this variation.

**Design, Setting, and Participants:**

This cross-sectional study used publicly available data from the Medicaid Drug Utilization Database from 2020 and 2021 and peer-reviewed data on NSCLC incidence, the prevalence of *EGFR* and *ALK* alterations, and expected treatment durations to estimate expected use of targeted therapies for *EGFR-* and *ALK-*altered NSCLC in 33 states.

**Exposures:**

State-specific Medicaid programs and state policies and characteristics.

**Main Outcomes and Measures:**

The primary outcome was the estimated proportion of person-time of Medicaid patients with *EGFR*- or *ALK-*altered NSCLC associated with receipt of targeted therapy in each state Medicaid program. Nested linear regression models examined associations between the observed variation and state policies and characteristics.

**Results:**

There were an estimated 3461 person-years in which *EGFR-* and *ALK-*targeted therapies were indicated in 2020 and 2021. During these years, only 2281 person-years of *EGFR*- and *ALK*-targeted therapies were dispensed to Medicaid patients, suggesting that an estimated 66% of Medicaid patients with *EGFR*- and *ALK*-altered metastatic disease received indicated targeted therapies across all states. Rates of targeted therapy use ranged from 18% in Arkansas to 113% in Massachusetts; 30 of 33 states (91%) had lower rates of targeted therapy use than expected. The observed variation across state Medicaid programs was associated with Medicaid policies, the density of oncologists, and state gross domestic product per capita.

**Conclusions and Relevance:**

This study suggests that rates of targeted therapy use among Medicaid patients with *EGFR-* and *ALK-*altered NSCLC were lower than expected and varied across state Medicaid programs. State policies and characteristics were associated with the observed variation, indicating where interventions could improve access to treatment and outcomes for patients with NSCLC.

## Key Points

**Question:**

Does the use of targeted therapies for non–small cell lung cancer (NSCLC) vary across state Medicaid programs?

**Findings:**

This cross-sectional study found substantial variation in the use of targeted therapies for *EGFR*- and *ALK-*altered NSCLC across Medicaid programs, with evidence of underuse in 30 of 33 states. The observed variation was associated with Medicaid policies, oncologist density, and state gross domestic product per capita.

**Meaning:**

This study suggests that targeted therapies are underused in many state Medicaid programs, limiting access to efficacious treatments; attention is needed for state policies and characteristics that may limit access to these useful drugs.

## Teaser

This cross-sectional study characterizes state-level variation in the use of targeted therapies among Medicaid patients with metastatic non–small cell lung cancer and describes factors associated with this variation.

## Introduction

Novel medications targeting genomic alterations in non–small cell lung cancer (NSCLC) have substantially improved overall survival for some patients. *EGFR* (OMIM 131550) variants and *ALK* (OMIM 105590) rearrangements are the 2 most common targetable alterations in NSCLC; as of 2020, eight targeted therapies were approved by the US Food and Drug Administration (FDA) to treat patients with tumors harboring these alterations. These medications are rarely used to treat malignant neoplasms other than NSCLC. Osimertinib, which targets *EGFR*, and alectinib, which targets *ALK*, have been the standard of care first-line treatments since 2018 for patients with these alterations.^1^

In the FLAURA trial, patients with metastatic *EGFR-*altered NSCLC treated with first-line osimertinib had a median overall survival of 38.6 months (95% CI, 34.5-41.8 months),^2^ leading the FDA to approve osimertinib as first-line therapy in 2018. In the ALEX trial, patients with metastatic NSCLC with *ALK* rearrangements treated with alectinib had a 5-year survival rate of 62%, and alectinib was approved by the FDA as first-line therapy in 2017.^3^ By contrast, median overall survival for patients with NSCLC who receive chemoimmunotherapy, the standard of care when targetable variants are not discovered, is 22 months.^4^

Prior work has shown that use of *EGFR-* and *ALK-*targeted therapies was lower than expected^5,6,7^; being insured by Medicaid, the state-administered health insurance program for the poor, was associated with lower use of targeted therapies.^8^ Effective drugs are underused in the US for many reasons, one of the most important being cost.^9^ Osimertinib and alectinib have list prices of more than $150 000 per year of treatment, and they are usually taken for several years, until disease progression or intolerable adverse effects occur.

High-cost novel drugs are a particular concern for state Medicaid programs because these programs have fixed overall budgets and are required by federal law to cover drugs for nearly all indications approved by the FDA. Fiscal concerns have led some Medicaid programs to limit patient enrollment, reduce the services provided, and implement utilization management strategies for costly drugs.^10,11^ The latter include prior authorization, which requires prescribers to justify their use of a high-priced drug before a prescription can be filled, copayments, and limitations on prescribing doses and durations. Utilization management programs reduce prescribing of expensive drugs, even for highly efficacious treatments.^12,13^ Patients insured by Medicaid also face challenges accessing subspecialist oncologists and receive lower rates of guideline-concordant care.^14,15^

Previous studies have shown that prescribing of costly drugs differs widely across state Medicaid programs^13^ and that access to evidence-based oncology care for Medicaid patients varies geographically.^16,17^ However, descriptions of the patterns of use of novel oncology therapies across state Medicaid programs are currently lacking. We aimed to characterize state-level variation in the use of targeted therapies among Medicaid patients with metastatic NSCLC and to describe factors that may be associated with this variation.

## Methods

In this cross-sectional study, we estimated appropriate use of targeted therapies as the proportion of person-time of Medicaid patients with *EGFR-* and *ALK-*altered metastatic NSCLC treated with targeted therapy in 2020 and 2021, based on (1) the number of person-years of *EGFR-* and *ALK*-targeted therapies dispensed within each state Medicaid program compared with (2) the expected person-years of such treatment in each state Medicaid population. The study was approved by the Mass General Brigham institutional review board. Informed consent was not required because all data were aggregated and deidentified. This study followed the Strengthening the Reporting of Observational Studies in Epidemiology (STROBE) reporting guideline.^18^

### Data Sources and Population

We obtained prescription data from the Medicaid Drug Utilization Database (MDUD)—quarterly state-specific aggregated data published by the Centers for Medicare & Medicaid Services. For national estimates, we included data from all 50 states and Washington DC. For state analyses, we included states with at least 20 estimated person-years of first-line *EGFR*-targeted therapy for metastatic NSCLC. We estimated the volume of prescriptions from 340B entities—health care organizations receiving discounted prices for outpatient medications through the federal 340B Drug Pricing Program—using IQVIA’s Longitudinal Access and Adjudication Data Set.^19,20^ To estimate the person-years of expected *EGFR-* and *ALK-*targeted therapy in each state’s Medicaid population, we combined demographic data from the 2019 American Community Survey,^21^ Medicaid enrollment reports for July of 2020 and 2021,^22^ age-specific incidences of NSCLC from registry data,^23^ and race- and ethnicity-specific prevalences of *EGFR* variants among Asian,^24^ Hawaiian or Pacific Islander,^25^ Hispanic,^26^ Black, and White non-Hispanic patients.^27^

Smoking rates and state gross domestic product (GDP) per capita were obtained from the Centers for Disease Control and Prevention and US Department of Commerce, respectively.^28,29^ Medicaid policy details were obtained from the Kaiser Family Foundation and review of Medicaid prescription drug formularies and fee schedules, completed between August 1, 2021, and April 30, 2022.^30^

### Calculating Targeted Therapy Use

To calculate the doses of *EGFR*- and *ALK*-targeted therapies dispensed, we used the MDUD to extract the number of prescriptions and units dispensed for all National Drug Codes for medications approved by the FDA as of January 1, 2020, to treat *EGFR-* or *ALK*-altered NSCLC (eTable 1 in Supplement 1). We included all calendar quarters from January 1, 2020, through December 31, 2021. The number of units dispensed for each drug was aggregated across the study period, and the number of person-years of treatment was estimated based on the full-strength dose in the FDA-approved labeling. For state-level analyses, we limited the analysis to osimertinib and alectinib, the preferred first-line therapies for *EGFR-* and *ALK-*altered NSCLC. Due to federal privacy laws, the Centers for Medicare & Medicaid Services censored data in quarters with fewer than 11 prescriptions for a given National Drug Code in a state. For these records, we conservatively imputed 300 units dispensed (ie, ten 30-day prescriptions) for that National Drug Code during that quarter in that state.

Prescriptions filled at 340B entities should not be present in the MDUD because, to avoid duplicate discounts, 340B entities cannot bill state Medicaid programs for these medications.^31^ To account for this limitation, we estimated the number of claims for osimertinib and alectinib from 340B entities within each state’s Medicaid population using pharmacy claim volumes from IQVIA’s Longitudinal Access and Adjudication Data Set.^20^ We assigned prescriptions to 340B entities if the prescription was written at a 340B entity and filled at the entity’s outpatient or contract pharmacy.

### Estimating Expected Targeted Therapy Use

To estimate the expected number of person-years of first-line *EGFR-* and *ALK-*targeted therapies for patients with metastatic NSCLC, we first estimated the number of Medicaid patients in each age and race and ethnicity stratum within each state, as the products of the age and race and ethnicity proportions from the American Community Survey and the total Medicaid enrollment numbers for each state. We then estimated the number of incident cases of NSCLC as the product of the number of patients in each age stratum, multiplied by age-specific NSCLC incidence. We assumed age-specific NSCLC incidences were constant across race and ethnicity and the period of analysis. Medicaid patients aged 65 years or older were excluded because their medications are usually covered by Medicare. Incidence of NSCLC among individuals younger than 30 years was considered negligible.

Because the prevalence of *EGFR* variants varies by race and ethnicity, we estimated the number of incident cases with *EGFR-*altered metastatic NSCLC as the product of the number of incident NSCLC cases within each racial and ethnic group and the race- and ethnicity-specific prevalences of *EGFR* variants. For *ALK* rearrangements, we assumed a prevalence of 3% of metastatic NSCLC cases across all races and ethnicities. Data used for these estimates are listed in eTable 2 in Supplement 1. These incidences were multiplied by the expected duration of each first-line treatment to estimate the expected person-years of *EGFR*- and *ALK-*targeted first-line therapy if used fully. To estimate duration of treatment, we identified studies reviewing real-world use of osimertinib and other *EGFR* inhibitors,^32,33,34,35,36,37^ and compared that data with relevant clinical trial populations, to estimate the ratio of real-world treatment duration to clinical trial progression-free survival, which we multiplied by the progression-free survival from pivotal trials.^38,39,40^ To account for uncertainty of the race- and ethnicity-specific prevalences of *EGFR* variants, we conducted an analysis of extremes for each race- and ethnicity-specific prevalence (eTable 2 in Supplement 1).

### Statistical Analysis

We estimated the percentage of eligible patient-years covered by targeted therapy in each state and ranked the states based on these estimates. We then categorized states as those in which such use was (1) within a range of plausible clinical variation (“at expected levels”), (2) below the range of plausible variation but greater than 50% of expected use (“somewhat below expected levels”), and (3) below 50% of expected use (“substantially below expected levels”).

We then performed sensitivity analyses excluding the imputed dispensings for censored data and 340B prescriptions. To assess whether these results were owing to demographic adjustments, we also conducted sensitivity analyses comparing the number of doses of osimertinib and alectinib administered and the number of Medicaid beneficiaries aged 30 to 65 years. To assess consistency of results across sensitivity analyses, we used Pearson correlation coefficients between the rankings of states in the main analysis and in each sensitivity analysis. We also assessed the stability of rankings by calculating the proportion of states where rankings moved by more than 5 places in each sensitivity analysis.

Finally, we used nested multivariable linear regression models to evaluate whether state characteristics or state Medicaid policies were associated with the proportion of eligible person-time covered by targeted treatment. State Medicaid policies that could be associated with access to osimertinib and alectinib were combined into a composite “access score” for each state. States were given a score of 0 to 4, with 1 point assigned for each of the following: no copayment requirements for branded medications, no prior authorization requirements for osimertinib, adequate coverage for *EGFR* testing (*Current Procedural Terminology* code 81235), and expansion of Medicaid under the Patient Protection and Affordable Care Act before 2020. The access score, the number of oncologists per 100 000 Medicaid beneficiaries, and state GDP per capita in 2020 were sequentially added to the model based on hypothesized causal mechanisms (eFigure 1 in Supplement 1). Smoking rates were not included because of collinearity with state GDP per capita. Statistical tests were 2-sided, and *P* < .05 was considered statistically significant. Analyses were performed using Stata, version 17.1 (StataCorp LLC).

## Results

We found that 2281 person-years of *EGFR*- and *ALK*-targeted therapy were dispensed in all state Medicaid programs in 2020 and 2021 (Table 1). Using pooled real-world treatment durations of 21.9 months for osimertinib and 40.6 months for alectinib (eFigure 2 in Supplement 1), we estimated there were 3461 person-years of expected first-line treatment with *EGFR-* and *ALK-*targeted therapies during this time. Thus, 66% of the person-time of Medicaid beneficiaries with *EGFR-* and *ALK-*altered metastatic NSCLC was associated with receipt of targeted therapy. Osimertinib and alectinib accounted for 83% of prescriptions (1901 person-years) for *EGFR*- and *ALK*-targeted therapy (Table 1).

**Table 1.** Total Person-Years of *EGFR*- and *ALK*-Targeted Therapies Dispensed Across All State Medicaid Programs in 2020 and 2021^a^

Therapy	No. of person-years^b^	
	2020	2021
Osimertinib	553	583
Alectinib	385	380
Crizotinib	79	56
Lorlatinib	45	58
Other TKIs^c^	100	40
Total^d^	1163	1118

Abbreviation: TKIs, tyrosine kinase inhibitors.

^a^ All state Medicaid programs include the 50 states and Washington, DC.

^b^ Person-years dispensed represents the number of years of full-strength treatment, as indicated on US Food and Drug Administration–approved labeling, dispensed for each medication.

^c^ Other tyrosine kinase inhibitors include afatinib, brigatinib, ceritinib, dacomitinib, erlotinib, and gefitinib.

^d^ Discrepancies in total numbers are because the person-years for each therapy have been rounded.

**Figure 1.**
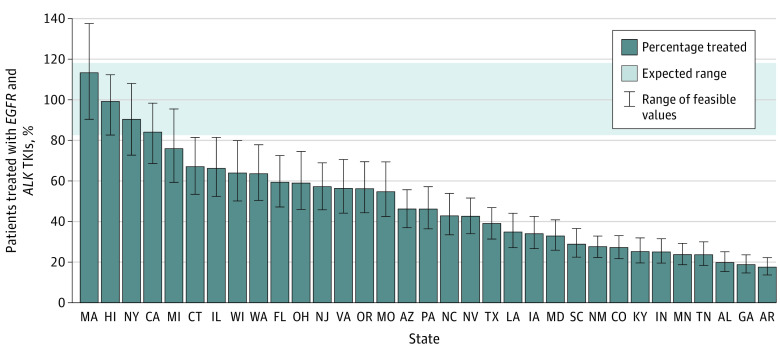
Osimertinib or Alectinib Use by State Medicaid Programs, Compared With Expected Levels of Use, 2020-2021 Estimated percentage of person-time with *EGFR*- and *ALK*-altered non–small cell lung cancer within each state Medicaid program that was associated with treatment with osimertinib or alectinib. The gray area represents the range of expected use based on the range of treatment durations. The error bars represent the range of possible percentages in each state based on the maximum and minimum feasible estimates of race- and ethnicity-specific prevalences of *EGFR* and *ALK* alterations. TKI indicates tyrosine kinase inhibitor.

**Figure 2.**
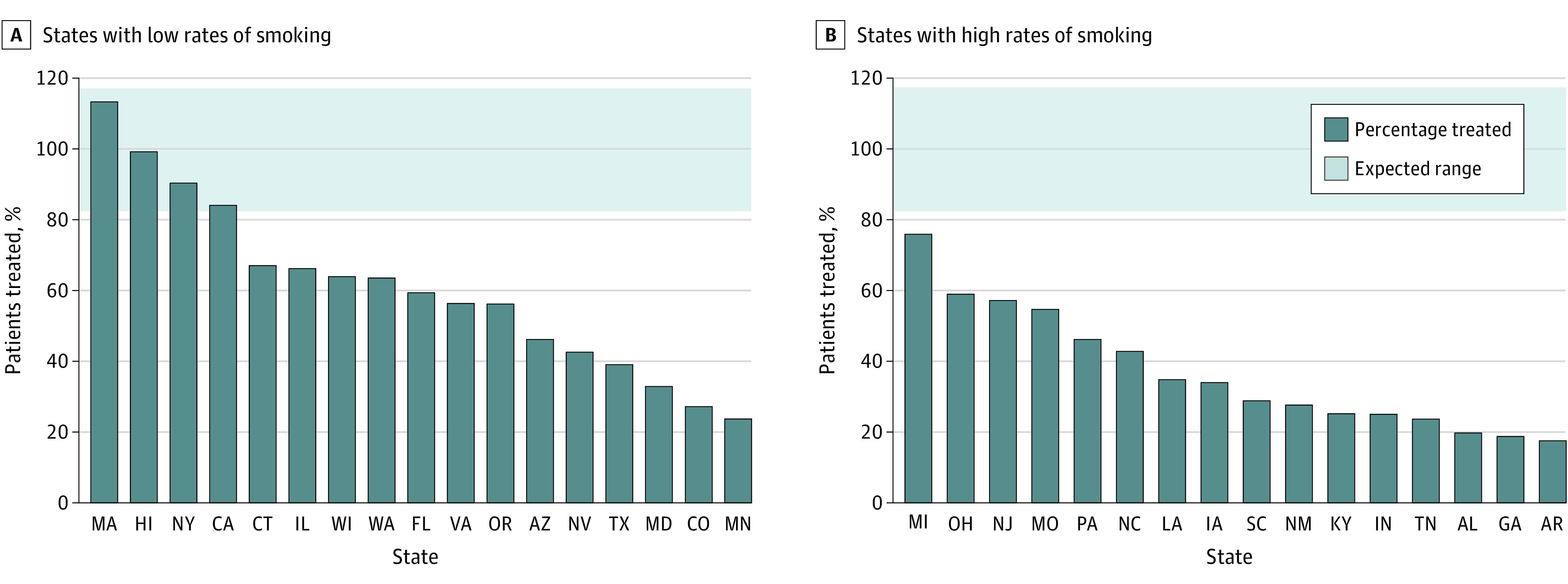
Actual vs Expected Osimertinib or Alectinib Use in State Medicaid Programs (2020-2021), Stratified by Smoking Rates A, States with low rates (<16%) of smoking among adults. B, States with high rates (≥16%) of smoking among adults. Estimated percentage of person-time with *EGFR*- and *ALK*-altered non–small cell lung cancer within each state Medicaid program that was associated with treatment with osimertinib or alectinib. The gray area represents the range of expected use based on the range of treatment durations.

Thirty-three states had at least 20 estimated person-years of *EGFR*-altered NSCLC during 2020 and 2021 and were included in the analysis (eFigure 3 in Supplement 1). These states accounted for 3258 person-years of expected *EGFR-* and *ALK-*targeted therapy use, vs 1991 person-years of osimertinib and alectinib use (eTable 3 in Supplement 1). The estimated proportion of person-time associated with osimertinib and alectinib use was 61% across these 33 states; the proportion in individual state Medicaid programs ranged from 18% in Arkansas to 113% in Massachusetts (Figure 1). Only 3 states (9%) had dispensing volumes consistent with expected levels, 12 states (36%) had dispensing volumes somewhat below expected levels, and 18 states (55%) had dispensing volumes substantially below expected levels. Large variations persisted after stratifying states by smoking rates (Figure 2): rates of expected use ranged from 18% to 76% in states with high smoking rates and 24% to 113% in states with low smoking rates. Pearson correlation coefficients were greater than 0.90 for all sensitivity analyses, and fewer than 3 states had rankings that changed more than 5 places in each analysis, indicating a high degree of consistency (eTable 4 in Supplement 1).

State Medicaid access scores—an ordinal measure of Medicaid “generosity”—are summarized in eTable 5 in Supplement 1. In regression analysis, each additional point of the Medicaid access score was associated with a 10.2% (95% CI, 1.3%-19.0%) increase in percentage of eligible days of osimertinib and alectinib use (Table 2). After adding the per-capita number of oncologists to the model, increasing access scores continued to be associated with increased targeted therapy use (13.2%; 95% CI, 5.0%-21.3%). The mean (SD) number of oncologists per 100 000 Medicaid enrollees (18.6 [6.4]), which ranged from 8.5 in New Mexico to 38.5 in Massachusetts, was associated with increased rates of targeted therapy use (1.6%; 95% CI, 0.4%-2.8%). When state GDP per capita was added to the models, state access scores and the number of oncologists were no longer associated with significant differences in use of targeted therapy. Increasing state GDP per capita was associated with increased rates of targeted therapy use in this model (0.9%; 95% CI, 0.1%-1.7%). The adjusted *R*^2^ of 0.40 indicates that the final model explained approximately 40% of the variation in targeted therapy use across states.

**Table 2.** Regression Results Showing Associations of Societal Variables With Alectinib or Osimertinib Use Among Medicaid Patients

Variable	% (95% CI)^a^		
	Model 1 (MAS)	Model 2 (MAS + OD)	Model 3 (MAS + OD + GDP)
Medicaid access score per point	10.2 (1.3 to 19.0)^b^	13.2 (5.0 to 21.3)^c^	7.1 (−2.0 to 16.3)
Oncologists per 100 000 Medicaid enrollees	NA	1.6 (0.4 to 2.8)^c^	0.8 (−0.6 to 2.2)
State GDP per capita, thousands	NA	NA	0.9 (0.1 to 1.7)^b^
Adjusted *R*^2^	0.12	0.31	0.40
Change in adjusted *R*^2^	NA	0.19	0.09

Abbreviations: GDP, gross domestic product; MAS, Medicaid access score; NA, not applicable; OD, oncologists per 100 000 Medicaid enrollees.

^a^ Coefficients from nested linear regression models assessing the associations of the dependent variables with rates of alectinib or osimertinib use among patients with *EGFR*- and *ALK*-altered non–small cell lung cancer. Each state was assigned a Medicaid access score of 0 to 4 based on the following criteria: no copayment requirements for branded medications (1 point), no prior authorization requirements for osimertinib and alectinib (1 point), adequate coverage for *EGFR* testing (1 point), and expanding Medicaid under the Patient Protection and Affordable Care Act in 2014 (1 point).

^b^ *P* < .05.

^c^ *P* < .01.

## Discussion

In analyzing the use of targeted therapies for metastatic *EGFR*- and *ALK*-altered NSCLC, we found substantial underprescribing of osimertinib and alectinib, with wide variation across state Medicaid programs. Just 66% of person-years in whom targeted therapies were indicated in 2020 and 2021 were associated with use of those medications, suggesting that at least 500 Medicaid patients with a diagnosis of *EGFR-* or *ALK-*altered metastatic NSCLC during these years did not receive targeted therapy when indicated. Given the efficacy of targeted therapies, this underuse could have led to an estimated 855 preventable years of life lost during the period of analysis.

The association between the observed variation and states’ Medicaid access scores suggests that state policies and characteristics are associated with use of these medications. For example, inadequate coverage of genomic testing may prevent oncologists from discovering which patients have *EGFR* and *ALK* alterations, a requirement to initiate osimertinib and alectinib in most states. Similarly, certain prior authorization processes may discourage the use of targeted therapies if oncologists do not have time to navigate the process or if patients with newly diagnosed cancer must start alternative treatment before prior authorizations are approved. Modifying state Medicaid policies may provide opportunities to improve access to life-prolonging treatments.

State GDP and the density of oncologists were also associated with the observed differences in multivariable models. State GDP, the dominant variable in the multivariable model, may be associated with access to these medications through changes in state budgets and policies or through community characteristics that are correlated with wealth, such as education, community resources, and smoking rates. The density of oncologists serves as a proxy for access to oncology care, which could mediate access to these medications. However, the density of oncologists does not necessarily represent access to oncologists for Medicaid patients, which can also be affected by coverage networks.^14^ The association between these characteristics and use of targeted therapies is important to understand; addressing them may require comprehensive policy solutions to address differential access to resources.

The adjusted *R*^2^ of 40% was substantial given the imprecise estimates of the outcome variable, indicating that the variables studied probably explain a reasonable amount of use of these targeted therapies across Medicaid programs. However, additional observable and unobservable factors may also be associated with access to targeted therapies. For example, the presence of a prior authorization requirement does not provide information about its level of complexity. Other observable factors such as the demographics of states’ Medicaid populations and rates of tobacco use may also be associated with rates of targetable variants and use of targeted therapies. Further research, including claims-based analyses at the level of individual patients, will be important to improve understanding of the factors associated with the observed variation in the use of these targeted therapies.

### Limitations

This study has some limitations. Without access to individual claims and medical records, we could not determine the exact number of patients with *EGFR-* and *ALK-*altered metastatic NSCLC, nor could we determine the number of patients who filled prescriptions for osimertinib and alectinib, as opposed to the aggregate use of these drugs. To produce these estimates, we used peer-reviewed estimates of NSCLC incidence, variant prevalence, and treatment durations. We also could not incorporate smoking rates into these estimates. Smoking rates are associated with the prevalence of *EGFR* and *ALK* alterations and, thus, with the expected use of targeted therapies, so differences in smoking rates across states may explain some of the differences seen here. To address these limitations, we conducted an analysis of extremes including all plausible epidemiologic values, and these results were robust to multiple sensitivity analyses. Considerable variation across states persisted after stratifying by smoking rates.

Our estimates assumed full-dose treatment during the entire course of treatment, which could result in underestimates of use. However, dose reductions are relatively uncommon with these medications, and rates of dose reductions are unlikely to vary between different states. Data suppression because of federal privacy laws prevented us from including less-frequently prescribed *EGFR-* and *ALK-*targeted therapies in the state-level analysis, which may have led to underestimates of targeted therapy use. However, these medications collectively accounted for fewer than 17% of prescriptions for *EGFR-* and *ALK-*targeted therapy during the period of analysis, so their exclusion does not explain the observed variation. We also did not account for the use of adjuvant osimertinib (approved by the FDA in December 2020) or subsequent-line targeted therapy use in our estimates of expected use. These omissions may have led to overestimates of targeted therapy use rates.

Data suppression prevented us from including smaller states in this analysis, which may have prevented the detection of further state-level variation. Among the states included, data suppression may have resulted in underestimates of targeted therapy use in smaller states. However, we included only states estimated to have prevalent cases that would result in approximately 30 prescriptions of osimertinib per quarter, almost triple the threshold of suppression. In addition, our estimation models conservatively replaced all suppressed cells with the maximum possible number of prescriptions below the threshold, which likely overestimated the use of osimertinib and alectinib.

These estimates do not include prescriptions from several other sources available to Medicaid patients, such as local-level health insurance plans for individuals with low or no income, pharmaceutical-sponsored drug assistance programs, the Veterans Affairs health system, and Medicare. However, patients enrolled in local-level health insurance plans for individuals with low or no income are unlikely to be enrolled in Medicaid, and pharmaceutical-sponsored drug assistance programs generally require that patients are not eligible for Medicaid. Excluding patients over the age of 64 years mitigated the number of prescriptions filled through Medicare, but we could not estimate the number of patients below the age of 65 years who were dually enrolled in Medicaid and Medicare or received care in the Veterans Affairs health system.

## Conclusions

We found evidence of underuse of targeted therapies for NSCLC among Medicaid beneficiaries and substantial variation in use of these efficacious medications across states. Where underuse is confirmed, policy makers should examine prescribing programs and practices in states to ensure that patients who require these life-prolonging medications are able to access them.

## Notes

Supplement 1. **eTable 1.** National Drug Codes Used to Identify *EGFR* and *ALK* Targeted Therapies **eTable 2.** Incidence and Prevalences Used to Estimate Targeted Therapy Use **eTable 3.** Details of Corrections for 340B Prescriptions and Data Censoring **eTable 4.** Correlation Coefficients Between Main Analysis and Sensitivity Analyses **eTable 5.** Summary of State Medicaid Access Scores **eFigure 1.** Directed Acyclic Graph (DAG) Depicting Causal Relationships Between Independent Variables and the Outcome of Interest, the Use of Osimertinib and Alectinib **eFigure 2.** Pooled Estimates of Real-World Treatment Duration **eFigure 3.** Number of Estimated Cases of *EGFR*-Mutated NSCLC by State in 2020 and 2021
eReferences

Supplement 2.
Data Sharing Statement
