# Supplementary material for: Variation in Use of Lung Cancer Targeted Therapies Across State Medicaid Programs, 2020-2021
Source: JAMA Netw Open. 2023 Jan 25;6(1):e2252562. doi: 10.1001/jamanetworkopen.2022.52562 (PMC10187487; doi:10.1001/jamanetworkopen.2022.52562)
Supplement: Supplement 1. — eTable 1. National Drug Codes Used to Identify EGFR and ALK Targeted Therapies eTable 2. Incidence and Prevalences Used to Estimate Targeted Therapy Use eTable 3. Details of Corrections for 340B Prescriptions and Data Censoring eTable 4. Correlation Coefficients Between Main Analysis and Sensitivity Analyses eTable 5. Summary of State Medicaid Access Scores eFigure 1. Directed Acyclic Graph (DAG) Depicting Causal Relationships Between Independent Variables and the Outcome of Interest, the Use of Osimertinib and Alectinib eFigure 2. Pooled Estimates of Real-World Treatment Duration eFigure 3. Number of Estimated Cases of EGFR-Mutated NSCLC by State in 2020 and 2021 eReferences [file jamanetwopen-e2252562-s001.pdf]

Supplementary Online Content

Roberts TJ, Kesselheim AS, Avorn J. Variation in use of lung cancer targeted therapies across state Medicaid programs, 2020-2021. *JAMA Netw Open.* 2023;6(1):e2252562. doi:10.1001/jamanetworkopen.2022.52562

eTable 1. National Drug Codes Used to Identify *EGFR* and *ALK* Targeted Therapies

eTable 2. Incidence and Prevalences Used to Estimate Targeted Therapy Use

eTable 3. Details of Corrections for 340B Prescriptions and Data Censoring

eTable 4. Correlation Coefficients Between Main Analysis and Sensitivity Analyses

eTable 5. Summary of State Medicaid Access Scores

eFigure 1. Directed Acyclic Graph (DAG) Depicting Causal Relationships Between Independent Variables and the Outcome of Interest, the Use of Osimertinib and Alectinib

eFigure 2. Pooled Estimates of Real-World Treatment Duration

eFigure 3. Number of Estimated Cases of *EGFR*-Mutated NSCLC by State in 2020 and 2021

eReferences

This supplementary material has been provided by the authors to give readers additional information about their work.

eTable 1. National Drug Codes Used to Identify *EGFR* and *ALK* Targeted Therapies

Generic Name	Proprietary Name	NDC
Afatinib	Gilotrif	00597013730
Afatinib	Gilotrif	00597013830
Afatinib	Gilotrif	00597014130
Gefitinib	Iressa	00310048230
alectinib	ALECENSA	50242013001
brigatinib	Alunbrig	63020009007
brigatinib	Alunbrig	63020009030
brigatinib	Alunbrig	63020011330
brigatinib	Alunbrig	63020018030
brigatinib	Alunbrig	63020019830
brigatinib	Alunbrig	76189011318
brigatinib	Alunbrig	76189011321
ceritinib	Zykadia	00078064070
ceritinib	Zykadia	00078069484
crizotinib	Xalkori	00069814020
crizotinib	Xalkori	00069814120
erlotinib	ERLOTINIB HYDROCHLORIDE	00093766356
erlotinib	ERLOTINIB HYDROCHLORIDE	00093766456
erlotinib	ERLOTINIB HYDROCHLORIDE	00378713193
erlotinib	ERLOTINIB HYDROCHLORIDE	00378713293
erlotinib	ERLOTINIB HYDROCHLORIDE	00378713393
erlotinib	ERLOTINIB HYDROCHLORIDE	42292005105
erlotinib	ERLOTINIB HYDROCHLORIDE	42292005305
erlotinib	ERLOTINIB HYDROCHLORIDE	50242006201
erlotinib	ERLOTINIB HYDROCHLORIDE	50242006301
erlotinib	ERLOTINIB HYDROCHLORIDE	50242006401
erlotinib	ERLOTINIB HYDROCHLORIDE	51991089033
erlotinib	ERLOTINIB HYDROCHLORIDE	51991089133
erlotinib	ERLOTINIB HYDROCHLORIDE	51991089233
erlotinib	ERLOTINIB HYDROCHLORIDE	63304009530
erlotinib	ERLOTINIB HYDROCHLORIDE	63304009630
erlotinib	ERLOTINIB HYDROCHLORIDE	63304013530
erlotinib	ERLOTINIB HYDROCHLORIDE	68382091306
erlotinib	ERLOTINIB HYDROCHLORIDE	68382091406
erlotinib	ERLOTINIB HYDROCHLORIDE	68382091506
erlotinib	ERLOTINIB HYDROCHLORIDE	72485021730
erlotinib	ERLOTINIB HYDROCHLORIDE	72485021830
erlotinib	ERLOTINIB HYDROCHLORIDE	72485021930
lorlatinib	Lobrena	00069022701
lorlatinib	Lobrena	00069023101
osimertinib	Tagrisso	00310134930
osimertinib	Tagrisso	00310135030

© 2023 Roberts TJ et al. *JAMA Network Open.*

eTable 2. Incidence and Prevalence Values Used to Estimate Targeted Therapy Use

Measure	Value	Minimum plausible	Maximum plausible
Age-specific NSCLC incidence (all stages)[1]			
30-49 years	2.6 cases / 100,000	-	-
50-64 years	75.9 cases / 100,000	-	-
Proportion of NSCLC cases metastatic (Stage IV) at diagnosis[1]	50.3%	-	-
Race-specific *EGFR* mutation prevalences			
Asian[2]	38%	33%	43%
Hawaiian / Pacific Islander[3]	38%	33%	43%
Hispanic[4]	25%	20%	30%
Other races[5]	15%	10%	20%
Prevalence of ALK mutations (all races)	3%	-	-

Sources of estimates are shown below in references. Estimates of treatment duration are shown in eFigure 2. 'Other races' includes all races and ethnicities in which the prevalence of EGFR mutation is not known to vary from population averages, including African American/Black and White.

© 2023 Roberts TJ et al. *JAMA Network Open.*

eTable 3. Details of Corrections for 340B Prescriptions and Data Censoring

A) Unadjusted estimated percentage of person-years treated in each state and the estimates after adjusting for 340B prescriptions and data censoring

state	Unadjusted person-years	person-years adjusted for 340B prescriptions	Person-years adjusted for 340B and censoring	Estimated person-years of treatment expected	Percentage of expected person-years associated with treatment (after adjustments)
**AL**	0.0	0.0	6.6	33.3	**19.8%**
**AR**	0.0	0.0	6.6	37.5	**17.6%**
**AZ**	42.8	42.8	42.8	92.8	**46.2%**
**CA**	487.3	542.0	542.0	644.9	**84.1%**
**CO**	14.1	14.2	17.4	64.2	**27.2%**
**CT**	23.1	25.5	32.1	47.9	**67.0%**
**FL**	76.2	95.0	95.0	160.1	**59.4%**
**GA**	1.0	1.8	11.7	62.3	**18.8%**
**HI**	17.2	17.2	23.8	24.0	**99.2%**
**IA**	0.0	0.0	9.9	29.0	**34.0%**
**IL**	79.0	91.0	91.0	137.5	**66.2%**
**IN**	14.1	14.1	17.4	69.5	**25.0%**
**KY**	6.7	10.7	17.2	68.5	**25.2%**
**LA**	14.9	17.0	23.6	67.7	**34.8%**
**MA**	57.3	101.1	101.1	89.2	**113.3%**
**MD**	8.6	13.5	20.1	61.1	**32.9%**
**MI**	66.3	95.0	95.0	125.2	**75.9%**
**MN**	3.3	3.3	13.2	55.7	**23.7%**
**MO**	7.4	15.7	22.3	40.8	**54.7%**
**NC**	16.2	19.2	26.9	62.9	**42.8%**
**NJ**	37.5	44.6	47.9	83.8	**57.2%**
**NM**	1.6	1.8	11.7	42.2	**27.6%**
**NV**	12.0	12.3	15.6	36.6	**42.6%**
**NY**	275.4	326.8	326.8	361.7	**90.3%**
**OH**	64.4	75.4	75.4	127.9	**58.9%**
**OR**	28.4	28.4	28.4	50.5	**56.2%**
**PA**	65.4	73.1	73.1	158.3	**46.2%**
**SC**	0.7	0.7	10.6	36.6	**28.8%**
**TN**	0.0	0.0	13.2	55.6	**23.7%**
**TX**	41.4	54.4	54.4	139.4	**39.0%**
**VA**	28.4	29.3	32.6	57.8	**56.3%**
**WA**	47.0	50.8	50.8	79.9	**63.5%**
**WI**	22.0	34.8	34.8	54.4	**63.9%**

Person-years refers to the number of person years of prescriptions for osimertinib and alectinib in each state during 2020 and 2021. Adjustments for 340B and data censoring are described in the methods section. Estimated person-years of expected treatment refers to the number of estimated person-years eligible for first-line therapy for EGRF- *and* ALK-*targeted therapies.*

© 2023 Roberts TJ et al. *JAMA Network Open.*

eTable 4. Correlation Coefficients Between Main Analysis and Sensitivity Analyses

Sensitivity Analysis	Pearson's Correlation Coefficient
Excluding correction for prescriptions from 340B entities	0.95
Excluding correction for censored data	0.95
Doses full-strength osimertinib and alectinib per 100,000 Medicaid beneficiaries	0.98

© 2023 Roberts TJ et al. *JAMA Network Open.*

eTable 5. Summary of State Medicaid Access Scores

State	No prior authorization for osimertinib	No copay for branded medications (2019)	Adequate coverage for *EGFR* testing	Medicaid expansion (2014)	Medicaid Access Score
AL	1	0	0	0	**1**
AR	0	0	1	1	**2**
AZ	0	1	1	1	**3**
CA	0	1	1	1	**3**
CO	0	0	1	1	**2**
CT	1	1	1	1	**4**
FL	0	1	0	0	**1**
GA	0	0	0	0	**0**
HI	0	1	1	1	**3**
IA	0	0	1	1	**2**
IL	0	0	0	1	**1**
IN	0	0	1	1	**2**
KY	0	0	1	1	**2**
LA	1	0	1	1	**3**
MA	0	0	1	1	**2**
MD	0	0	1	1	**2**
MI	1	0	1	1	**3**
MN	0	0	1	1	**2**
MO	0	0	0	0	**0**
NC	1	0	0	0	**1**
NJ	0	1	1	1	**3**
NM	0	1	1	1	**3**
NV	0	1	0	1	**2**
NY	1	0	1	1	**3**
OH	0	0	1	1	**2**
OR	0	1	1	1	**3**
PA	0	0	1	1	**2**
SC	0	0	1	0	**1**
TN	1	0	0	0	**1**
TX	1	1	0	0	**2**
VA	0	0	1	1	**2**
WA	0	1	1	1	**3**
WI	1	0	1	0	**2**

eFigure 1. Directed Acyclic Graph (DAG) Depicting Causal Relationships Between Independent Variables and the Outcome of Interest, the Use of Osimertinib and Alectinib

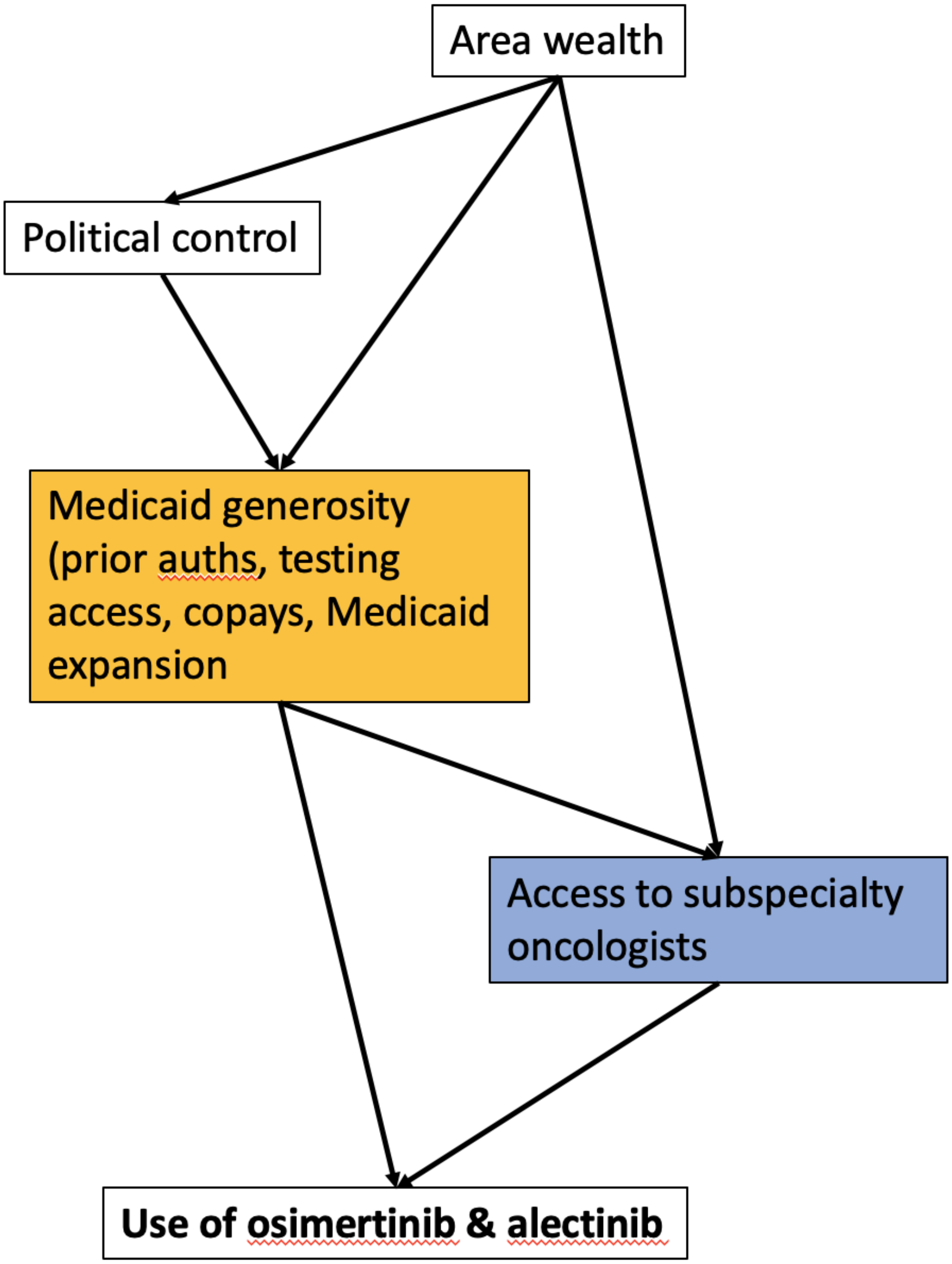

© 2023 Roberts TJ et al. *JAMA Network Open.*

eFigure 2. Pooled Estimates of Real-World Treatment Duration

A) Included studies evaluating real-world treatment duration with their estimated treatment duration and progression-free survival from relevant clinical trial populations

Real-World Study						Clinical Trial Comparison Group				
**Study**	**Description**	**Metric Reported**	**Sample size**	**Mean**	**95% CI**	**Relevant clinical trial**	**Intervention or control arm**	**Sample size**	**PFS**	**95% CI**
Singal et al.[8]	earlier generation EGFR TKIs	TTD	380	10.3	8.7-11.5	FLAURA[6]	control	277	10.2	9.6-11.1
Viray et al.[9]	first-line osimertinib	TTD	56	16.9	12.5-35.1	FLAURA[6]	intervention	279	18.9	15.2-21.4
Lorenzi et al.[10]	first-line osimertinib	TTD	126	21.4	11.8-30.9	FLAURA[6]	intervention	279	18.9	15.2-21.4
Ohe et al.[11]	subsequent-line osimertinib	PFS	3578	12.3	12.2-12.6	AURA3[12]	intervention	279	10.1	8.3-12.3
Imamura et al.[13]	subsequent-line osimertinib	PFS	147	17.2	11.9-22.5	AURA3[12]	intervention	279	10.1	8.3-12.3
Marinis et al.[14]	subsequent-line osimertinib	PFS	3015	13.5	12.6-13.9	AURA3[12]	intervention	279	10.1	8.3-12.3

TTD = total treatment duration, PFS = progression-free survival, CI = confidence interval; When real-world trials reported TTD and PFS, TTD was used.

B) Forest plot showing ratio of real-world treatment durations and clinical trial progression free survival for each study and the pooled ratio

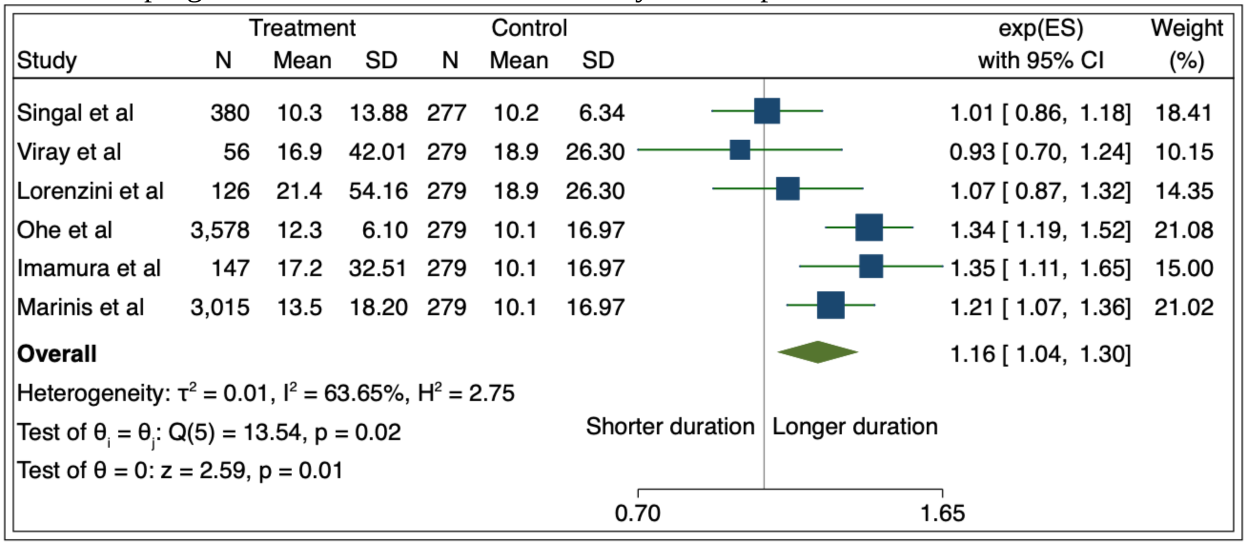

© 2023 Roberts TJ et al. *JAMA Network Open.*

C) Estimated treatment duration for first-line osimertinib and alectinib based on the pooled ratio of real-world to clinical trial treatment durations

Drug	Trial	Trial PFS	Ratio (95% CI)	Estimated Real-World Treatment Duration (95% CI)
Osimertinib	FLAURA[6]	18.9	1.16 (1.04 - 1.30)	21.9 (19.7 - 24.6)
Alectinib	ALEX[7]	35.0	1.16 (1.04 - 1.30)	40.6 (36.4 - 45.5)

PFS = progression-free survival; CI = confidence interval; Ratio refers to the ratio of clinical trial PFS to real-world treatment duration, which was calculated as the pooled estimate of ratios from 6 real-world trials (eFigure 2b); Estimated real-world treatment duration was calculated as the product of this ratio and the clinical trial PFS.

eFigure 3. Number of Estimated Cases of *EGFR*-Mutated NSCLC by State in 2020 and 2021

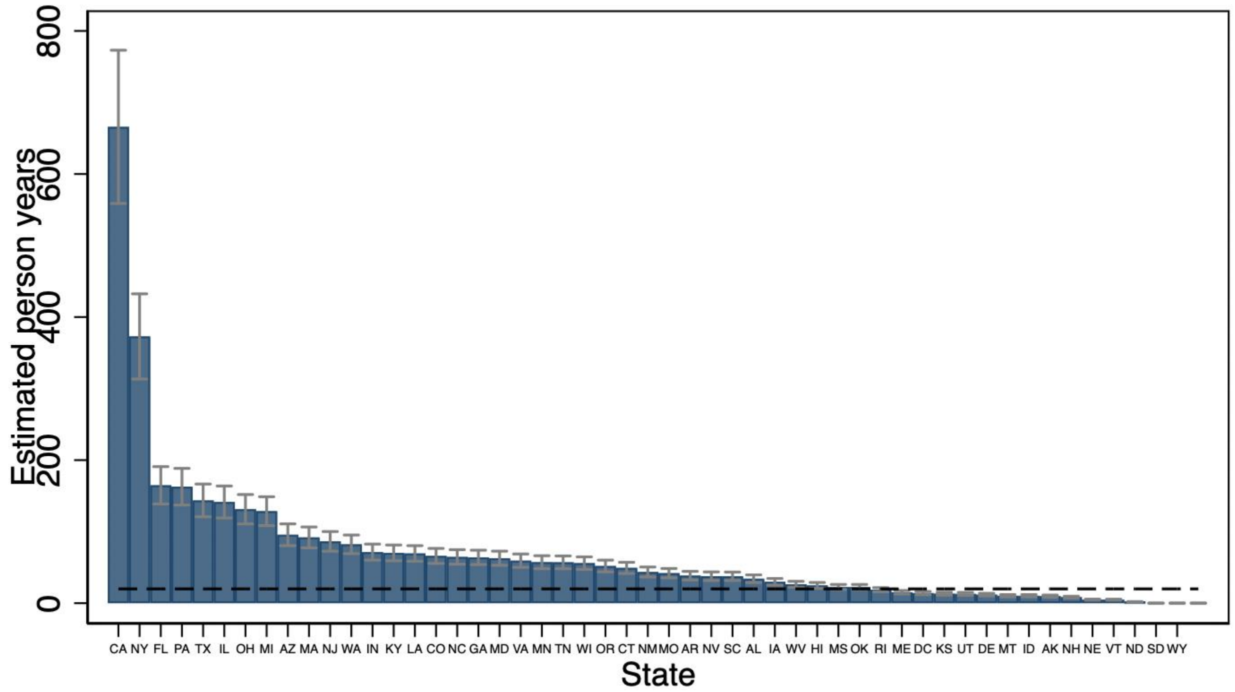

Estimated number of person-years in each state during 2020 and 2021

Twenty person-years (threshold for inclusion)

Range of possible person-years when using different race-specific prevalences of *EGFR* mutations

Number of person-years of EGFR*-mutated NSCLC were estimated as the product of the number of incident cases in 2020 and 2021 and the expected duration of treatment based on data from clinical trials. The number of incident cases of* EGFR*-mutated NSCLC in 2020 and 2021 was estimated based on registry data and race-specific prevalences of* EGFR *mutations. The gray bars represent the range of possible person-years when plausible race-specific prevalences of* EGFR *mutations were used.*

© 2023 Roberts TJ et al. *JAMA Network Open.*

eReferences

1. SEER*Explorer Application. Accessed August 31, 2021. https://seer.cancer.gov/explorer/application.html

2. Chen J, Yang H, Teo ASM, et al. Genomic landscape of lung adenocarcinoma in East Asians. *Nat Genet*. 2020;52(2):177-186. doi:10.1038/s41588-019-0569-6

3. Yatabe Y, Kerr KM, Utomo A, et al. EGFR mutation testing practices within the Asia Pacific region: results of a multicenter diagnostic survey. *J Thorac Oncol Off Publ Int Assoc Study Lung Cancer*. 2015;10(3):438-445. doi:10.1097/JTO.0000000000000422

4. Villena-Vargas J, Mynard N, Voza F, et al. P37.04 EGFR Mutations in US Hispanics with Lung Adenocarcinoma are Common and Portend a Worse Prognosis. *J Thorac Oncol*. 2021;16(3):S443-S444. doi:10.1016/j.jtho.2021.01.751

5. Campbell JD, Lathan C, Sholl L, et al. Comparison of Prevalence and Types of Mutations in Lung Cancers Among Black and White Populations. *JAMA Oncol*. 2017;3(6):801-809. doi:10.1001/jamaoncol.2016.6108

6. Soria JC, Ohe Y, Vansteenkiste J, et al. Osimertinib in Untreated EGFR-Mutated Advanced Non–Small-Cell Lung Cancer. *N Engl J Med*. 2018;378(2):113-125. doi:10.1056/NEJMoa1713137

7. Peters S, Camidge DR, Shaw AT, et al. Alectinib versus Crizotinib in Untreated ALK-Positive Non–Small-Cell Lung Cancer. *N Engl J Med*. 2017;377(9):829-838. doi:10.1056/NEJMoa1704795

8. Singal G, Miller PG, Agarwala V, et al. Association of Patient Characteristics and Tumor Genomics With Clinical Outcomes Among Patients With Non–Small Cell Lung Cancer Using a Clinicogenomic Database. *JAMA*. 2019;321(14):1391-1399. doi:10.1001/jama.2019.3241

9. Viray H, Piper-Vallillo A, Widick P, et al. A real-world study of patient characteristics and clinical outcomes in EGFR-mutated lung cancer treated with first-line osimertinib. *J Clin Oncol*. 2022;40(16_suppl):e21033-e21033. doi:10.1200/JCO.2022.40.16_suppl.e21033

10. Lorenzi M, Ferro A, Cecere F, et al. First-Line Osimertinib in Patients with EGFR-Mutant Advanced Non-Small Cell Lung Cancer: Outcome and Safety in the Real World: FLOWER Study. *The Oncologist*. n/a(n/a). doi:10.1002/onco.13951

11. Ohe Y, Kato T, Sakai F, et al. Real-world use of osimertinib for epidermal growth factor receptor T790M-positive non-small cell lung cancer in Japan. *Jpn J Clin Oncol*. 2020;50(8):909-919. doi:10.1093/jjco/hyaa067

12. Mok TS, Wu YL, Ahn MJ, et al. Osimertinib or Platinum–Pemetrexed in EGFR T790M–Positive Lung Cancer. *N Engl J Med*. 2017;376(7):629-640. doi:10.1056/NEJMoa1612674

13. Imamura F, Kimura M, Yano Y, et al. Real-world osimertinib for EGFR mutation-positive non-small-cell lung cancer with acquired T790M mutation. *Future Oncol*. 2020;16(21):1537-1547. doi:10.2217/fon-2020-0203

14. Marinis F de, Wu YL, de Castro G, et al. ASTRIS: a global real-world study of osimertinib in >3000 patients with EGFR T790M positive non-small-cell lung cancer. *Future Oncol*. 2019;15(26):3003-3014. doi:10.2217/fon-2019-0324

© 2023 Roberts TJ et al. *JAMA Network Open.*
