# Supplementary material for: Variation in Use of Lung Cancer Targeted Therapies Across State Medicaid Programs, 2020-2021
Source: JAMA Netw Open. 2023 Jan 25;6(1):e2252562. doi: 10.1001/jamanetworkopen.2022.52562 (PMC10187487; doi:10.1001/jamanetworkopen.2022.52562)
Supplement: Supplement 2. — Data Sharing Statement [file jamanetwopen-e2252562-s002.pdf]

Data Sharing Statement

Roberts. Variation in Use of Lung Cancer Targeted Therapies Across State Medicaid Programs, 2020-2021. *JAMA Netw Open*. Published January 25, 2023. doi:10.1001/jamanetworkopen.2022.52562

Data
Data available: Yes
Data types: Data (not involving human participants)
How to access data: All data are publicly available from the Center for Medicaid and Medicare Services: https://www.medicaid.gov/medicaid/prescription-drugs/state-drug-utilization-data/index.html
When available: With publication

Supporting Documents
Document types: None

Additional Information
Who can access the data: Data are publicly available.
Types of analyses: Data are publicly available for any purpose.
Mechanisms of data availability: Data are publicly available.
